# May the change of platelet to lymphocyte ratio be a prognostic factor for T3-T4 laryngeal squamous cell carcinoma: A retrospective study

**DOI:** 10.1371/journal.pone.0210033

**Published:** 2018-12-31

**Authors:** Bing Zhong, De-Ying Gu, Jin-Tao Du, Fei Chen, Ya-Feng Liu, Shi-Xi Liu

**Affiliations:** Department of Otorhinolaryngology Head and Neck Surgery, West China Hospital, Sichuan University, Chengdu, Sichuan province, China; Laurentian University, CANADA

## Abstract

**Background:**

Many blood markers have been shown to predict the recurrence and survival of various malignancies, but the effects of surgery on the body's inflammatory levels may cause changes in these inflammatory markers. Therefore, in this study, we assessed the relationship between changes in platelet to lymphocyte ratio (PLR) and survival and recurrence in patients with T3-T4 laryngeal squamous cell carcinoma (LSCC).

**Methods:**

Data of patients with T3-T4 HSCC were reviewed. Continuous variables were expressed as mean ± SD and were compared using t test or Mann-Whitney U test. The covariate distributions were compared by Chi-square test. Survival curve was estimated by Kaplan-Meier analysis, and Log-Rank test were performed to estimate the survival curve and significance of the difference in survival distribution between groups, respectively. The prognostic value was uncovered by univariate and multivariate Cox hazards analysis.

**Results:**

The 413 consecutive patients with LSCC were reviewed. Of these, 362 patients who met the criteria were selected, multi-factor analysis found that pathological T classification(hazard ratio [HR] = 1.878; 95% confidence interval [CI] = 1.342–3.023; P<0.001), pathological N classification (HR = 1.212; 95% CI = 0.867–2.125; P< 0.001) and change of PLR (HR = 2.158; 95% CI = 1.332–2.889; P = 0.004) associated with postoperative recurrence of T3-T4 LSCC. In addition, the pathological T classification (HR = 1.901; 95% CI = 1.255–2.999; P<0.001), pathological N classification (HR = 1.244; 95% CI = 0.810–2.212; P<0.001) and change of PLR (HR = 2.011; 95% CI = 1.354–2.753; P = 0.001) associated with postoperative survival in patients with T3-T4 LSCC.

**Conclusions:**

Results demonstrate that change in PLR may serve as a useful prognostic predictor for patients with T3-T4 LSCC.

## Introduction

LSCC is one of the most common tumors in the upper gastrointestinal tract LSCC, which accounts for about 1% to 5% of tumors[1, 2]. Smoking, harmful dust, poor oral hygiene, vitamin D metabolism disorder, endocrine disorder, and radiation or virus may lead to LSCC[3–6], which is most common in men or women aged 50–70 years[7, 8]. The incidence of LSCC in urban areas was higher than that in rural areas, and the incidence of LSCC in heavily polluted heavy industrial cities was higher than that in lightly polluted light industrial cities.

The mechanism of postoperative recurrence is complex and many factors act together. Inflammation plays a decisive role in the occurrence and development of many tumors[9, 10]. In recent years, it has been considered as an important factor affecting the recurrence and overall survival of LSCC in patients with laryngeal cancer, such as platelets, neutrophils[11]. Early treatment is often missed because people pay less attention to early symptoms such as hoarseness and many patients were diagnosed as T3-T4 LSCC at the time of the visit. Considering the extremely poor prognosis of T3-T4 LSCC, it is necessary to find a simple and effective prognostic index to judge the prognosis[12]. However, limited data on the prognostic effects of these indicators in LSCC patients may not be sufficient to accurately estimate the prognosis. In addition, the systemic inflammatory responses could be changed through surgery. In this study, we aim at that whether the change in PLR could serve as an indicator of mortality in patients with T3-T4 LSCC.

## Materials and methods

### Study group

This study was approved by the ethics committee of West China Hospital. All patients signed informed consent. All steps were conducted in accordance with the relevant principles. Patients identified with T3-T4 LSCC underwent tumor resection at West China Hospital (Chengdu, China) between 2007 and 2016 were enrolled in the retrospective study. We had access to information that could identify individual participants during or after data collection. LSCC was confirmed by postoperative pathology. Exclusion criteria are as follows: any other disease influencing blood cell lines including anemia, leukocyte disease, hemorrhagic disease and hematopoietic malignancy; patients who had been treated, including surgery, radiation and chemotherapy, both in other hospitals and in our hospital, regardless of the length of time interval of this surgery; patients who had history of the use of anticoagulant drugs; patients who discontinued treatment or were treated outside our hospital; lack of pretreatment examination of routine complete blood counts and blood biochemistry; patients with symptoms and signs of hepatic function damage that may influence PLR. We screen patients strictly according to inclusion and exclusion criteria to reduce bias.

### Follow-up and definitions

All patients underwent blood routine, blood biochemistry, coagulation, laryngoscopy, abdominal ultrasound, cervical computed tomography or magnetic resonance imaging and chest radiography before surgery. Blood tests were performed on day of admission and the day before surgery. The first follow-up was conducted in the first month after surgery, followed by every three months in the subsequent years. The last follow-up date was 83rd month after surgery. The postoperative projects were blood routine, blood biochemistry, laryngoscopy, neck and chest CT, abdominal ultrasound. Postoperative recurrence was defined as the pathological examination of laryngoscope biopsy or surgical resection. OS is defined as the time from the surgery to the time of death from any cause. RFS is defined as the time from the surgery to the time of recurrence. NLR is defined as absolute neutrophils count divided by lymphocyte count (109/L). PLR is defined as absolute platelet count divided by lymphocyte count (109/L). Preoperative PLR is defined as the average of two preoperative PLR. Postoperative PLR is defined as the average of PLR detected in the first three postoperative follow-up. PLR increase and decrease were defined as postoperative PLR minus preoperative PLR was > 0 and postoperative PLR minus preoperative PLR < 0, respectively.

### Statistical analysis

SPSS21 (SPSS Inc., Chicago, IL, USA) software was used for statistical analysis. Continuous variables were presented as the mean ± SD and compared by using the t-test for normal distribution and Mann-Whitney U test for abnormal distribution. Chi-square test is used to compare the covariate distribution. Survival curve was estimated by Kaplan-Meier analysis, and Log-Rank test was performed to test the significance of the difference in survival distribution between groups. Univariate and multivariate Cox hazards analyses were used to evaluate the relative effects of change of PLR on overall survival (OS) and (RFS). P < 0.05 was considered statistically significant.

## Results

A total of 477 consecutive patients with LSCC were reviewed, and 362 patients were enrolled in this study, including 39 female and 323 male. The mean age of patients was 49.1±12.2 years. The differentiation of well, moderate, and poor was detected in 137, 109 and 116 patients, respectively. 183 patients were diagnosed with T3 classification, whereas 179 were diagnosed with the T4 classification. In addition, 112, 126 and 124 patients had experienced the total, subtotal, and partial laryngectomy, respectively. The negative and positive surgical margins happened in 336 and 26 patients. 195 patients had increased PLR after surgery and 167 patients suffering from decreased PLR. During follow-up, 185 patients suffered the recurrence, while 191 patients died(Table 1).

**Table 1:** Patients’ characteristics of the study.

Variables	No./mean±SD
Age, y	49.1±12.2
Famale/male	39/323
Primary site (supraglottic/glottic/subglottic)	76/231/55
Differentiation (well/moderate/poor)	137/109/116
Pathological T classification (T3/T4)	183/179
Pathological N stage(Negative/Postive)	156/206
laryngectomy(Total/ Subtotal/ Partial)	112/126/124
Surgery margins (Negative/Postive)	336/26
Platelet, 10^9^ cells/L	113.3±52.8
Preoperative NLR	1.39±1.01
Preoperative PLR	88.4±54.1
Preoperative PNI	67.2±26.7
Preoperative NLR	1.42± 1.
Postoperative NLR	1.41±1.11
Postoperative PLR	87.2±33.4
Postoperative PNI	67.6±27.9
Change of PLR (increase vs. decrease)	195/167

**Fig 1:**
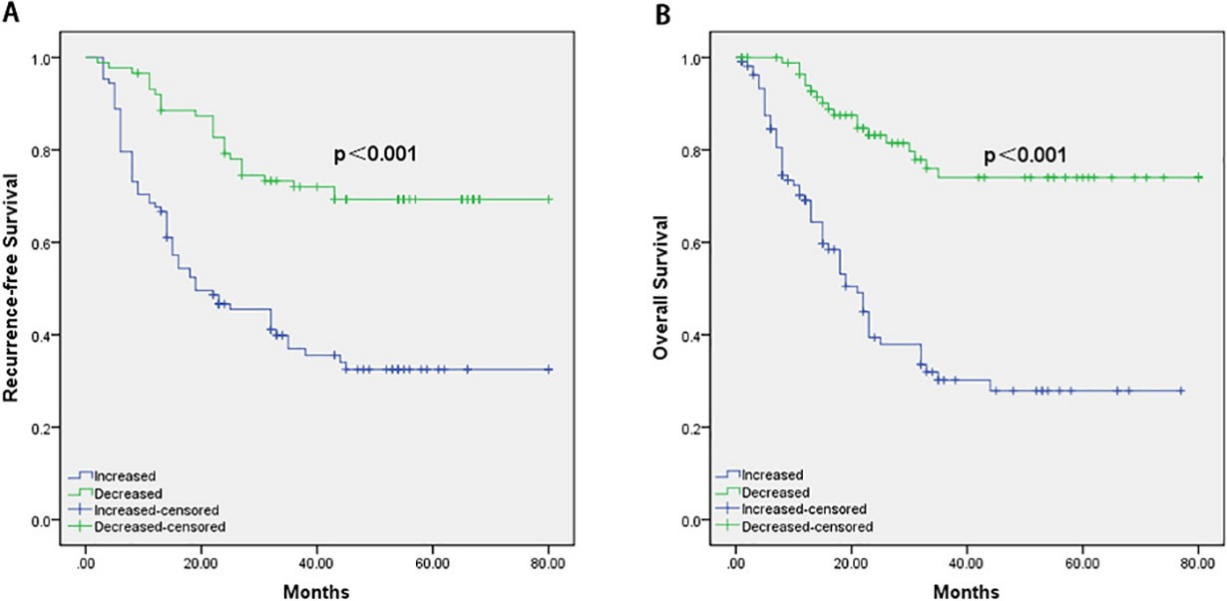
RFS and OS curves of patients. The increased postoperative PLR and decreased postoperative PLR of RFS(A) and OS(B).

Univariate analyses (Table 2) showed that RFS is associated with the pathological T classification, pathological N classification, and change of PLR. Multivariate analyses showed that pathological T classification (HR = 1.878; 95% CI = 1.342–3.023; P<0.001), pathological N classification (HR = 1.212; 95% CI = 0.867–2.125; P<0.001) and change of PLR (HR = 2.158; 95% CI = 1.332–2.889; P = 0.003) were independent risk factors for a worsened RFS(Table 3). The 5-year RFS rates of patients with increased PLR was 32.5%, whereas those of patients with decreased postoperative PLR was 68.4% (Fig 1A, P<0.001).

**Table 2:** Factors associated with postoperative recurrence in the univariate analyses.

Variables	Recurrence(185)	Nonrecurrence(177)	P
Age,y	47.2±10.5	51.7±13.5	0.554
Famale/male	19/166	20/157	0.752
Primary site (supraglottic/glottic/subglottic)	39/116/30	35/115/25	0.820
Differentiation (well/moderate/poor)	65/58/62	72/51/54	0.554
Pathological T classification (T3/T4)	66/119	117/60	<0.001
Pathological N classification (Negative/Postive)	58/127	98/79	<0.001
laryngectomy(Total/ Subtotal/ Partial)	55/67/63	57/59/61	0.819
Surgery margins (Negative/Postive)	171/14	165/12	0.772
Platelet, 10^9^ cells/L	112.7±53.4	114.8±53.9	0.441
Preoperative NLR	1.47±1.03	1.39±1.35	0.563
Preoperative PLR	89.5±52.1	87.1±58.2	0.554
Preoperative PNI	68.4±29.3	65.5±23.8	0.442
Postoperative NLR	1.64±1.54	1.58±1.18	0.741
Postoperative PLR	89.2±35.2	86.5±31.2	0.113
Postoperative PNI	68.9±29.2	66.7±24.5	0.669
Change of PLR (increase vs. decrease)	114/71	81/96	0.002

**Table 3:** Factors associated with postoperative recurrence in the multivariate analyses.

Variables	HR	95%CI	P
Pathological T classification	1.194	0.734–3.511	<0.001
Pathological N classification	1.531	0.747–2.421	<0.001
Change of PLR	1.684	1.149–2.943	0.003

Univariate analyses (Table 4) showed that pathological T classification, pathological N classification, and change of PLR were associated with OS after tumor resection. Pathological T classification (HR = 1.901; 95% CI = 1.255–2.999; P<0.001), pathological N classification (HR = 1.244; 95% CI = 0.810–2.212; P<0.001) and change of PLR (HR = 2.011; 95% CI = 1.354–2.753; P = 0.001) were demonstrated as independent prognostic factors for poor OS rate in the multivariate analysis (Table 5). The 5-year OS rate was 41.6% in patients with increased PLR and 71.4%, correspondingly, in patients with decreased PLR (Fig 1B, P<0.001).

**Table 4:** Factors associated with postoperative survival in the univariate analyses.

Variables	Survival(171)	Died(191)	P
Age, y	46.8±11.7	51.2±12.8	0.466
Famale/male	17/154	22/169	0.629
Primary site (supraglottic/glottic/subglottic)	33/112/26	43/119/29	0.745
Differentiation (well/moderate/poor)	61/58/52	76/51/64	0.327
Pathological T classification (T3/T4)	103/68	80/111	0.001
Pathological N classification (Negative/Postive)	91/80	65/126	<0.001
laryngectomy(Total/ Subtotal/ Partial)	51/64/56	61/62/68	0.611
Surgery margins (Negative/Postive)	161/10	175/16	0.352
Platelet, 10^9^ cells/L	111.9±53.8	114.2±54.1	0.509
Preoperative NLR	1.46±1.12	1.38±1.01	0.592
Preoperative PLR	90.1±53.7	87.5±58.7	0.755
Preoperative PNI	69.3±28.5	65.1±21.9	0.561
Postoperative NLR	1.67±1.44	1.57±1.11	0.892
Postoperative PLR	90.1.2±33.6	85.4±30.9	0.267
Postoperative PNI	69.4±28.4	66.3±22.1	0.673
Change of PLR (increase vs. decrease)	65/106	130/61	<0.001

**Table 5:** Factors associated with postoperative survival in the multivariate analyses.

Variables	HR	95%CI	P
Pathological T classification	1.758	1.143–3.014	<0.001
Pathological N classification	1.435	0.681–2.537	<0.001
Change of PLR	2.327	1.422–2.916	0.001

## Discussion

LSCC is a kind of malignant tumor with hoarseness, dysphagia and pharyngeal foreign body sensation as the main symptoms. Because of the early symptoms are easy to be ignored by the patients, the number of patients with T3-T4 LSCC continues to increase. Despite surgery and postoperative radiotherapy and chemotherapy, the prognosis is still unsatisfactory. In this study, we found that PLR is an independent predictor of RFS and OS of LSCC.

Platelets have long been considered key effector cells for hemostasis while identifying and killing invading pathogens and releasing various mediators that regulate immune and endothelial responses. But many previous studies have suggested that platelets may also contribute to tumor migration and metastasis[13, 14]. Platelets protect tumor cells from immune responses and promote cancer-related clotting, thereby promoting tumor growth and development. In addition, platelets contain many proteins that regulate angiogenesis and secrete cytokines and growth factors, such as VEGF, platelet-derived growth factor (PDGF), and FGF, leading to the development of the tumor, including angiogenesis, cell migration and, proliferation[15–17]. Clinical findings also suggest that the higher the number of platelets, the worse the prognosis for cancer patients[18]. Lymphocytes are a special kind of white blood cells produced by lymphocytes. As an important part of host immune monitoring system, lymphocytes play an important role in tumor growth and migration. It is well known that lymphocytes are closely related to the outcome of cancer patients[19, 20]. Therefore, PLR may be a significant prognostic indicator for cancer patients.

As a marker of tumor patients, thrombosis is the product of platelets and various blood coagulation factors. Among various cytokines, IL-6 plays a key role in the formation of thrombosis[21, 22]. IL-6 stimulates the production of inflammatory cells, inhibits the amount of albumin and the activity of enzymes involved in the synthesis of albumin in the liver. In addition, IL-6 can also stimulate the differentiation of megakaryocytes into platelets in the bone marrow and increase the number of platelets in the body[23, 24]. In fact, the vascular endothelial growth factor secreted by tumor cells also promotes platelet interactions with tumor cells, thereby accelerating tumor proliferation and metastasis[25].

PLR is associated with the prognosis of many cancer patients, including liver cancer, endometrial cancer, and rectal cancer[26, 27]. Interestingly, PLR has also been shown to be a valuable prognostic marker for heart failure and cerebral hemorrhage[28, 29]. PLR has been reported to be associated with the prognosis and mortality of patients with laryngeal cancer, which is also consistent with our study. Surgery can stimulate stress state of the body, resulting in the body including platelet, neutrophil, white blood cell changes, so that the inflammatory state of the patient changes. In addition, surgical bleeding and infection can also result in the release of various inflammatory factors and changes in the number of blood cells. The body responds to changes in physiological status after tumor resection through changes in the internal environment. Therefore, some changes in inflammatory markers after surgery may be more significant in predicting patients' prognosis. Preoperative and postoperative PLR have been reported as independent predictors of tumor patients, but it has been neglected that surgery as a hemorrhagic injury has a significant impact on systemic inflammatory levels. In this study, we considered the impact of surgery and confirmed that PLR changes were an independent predictor of the prognosis of T3-T4 LSCC. With the increase of PLR after surgery, the survival rate and recurrence rate of T3-T4 LSCC patients showed a significant upward trend. However, the decreased PLR showed higher RFS and OS.

This study has some limitations. First, this is a single-center retrospective study. Second, our study only involved patients with T3-T4 LSCC, whether this result is suitable for all LSCC needs further study.

## Conclusion

This study confirmed PLR's prediction of LSCC recurrence and mortality. The results show that the changes of PLR have strong predictive significance for the prognosis of LSCC. In fact, PLR, as a simple and obtained blood indicator, has significant significance in helping clinicians with stratified treatment and prognosis prediction.

## References

[1] Ahmadi N, Ahmadi N, Chan MV, Huo YR, Sritharan N, Chin R. Laryngeal Squamous Cell Carcinoma Survival in the Context of Human Papillomavirus: A Systematic Review and Meta-analysis. Cureus 2018; 10: e2234 10.7759/cureus.2234 29713579 PMC5919768

[2] Zhang H, Liu X, Chen L, Cai L, Li N, Zhu P, et al Differential expression of peroxiredoxin 3 in laryngeal squamous cell carcinoma. Oncotarget 2017; 8: 3471–3480. 10.18632/oncotarget.13838 27966448 PMC5356896

[3] Langevin SM, McClean MD, Michaud DS, Eliot M, Nelson HH, Kelsey KT. Occupational dustexposure and head and neck squamous cell carcinoma risk in a population-based case-control study conducted in the greater Boston area. Cancer Med 2013; 2: 978–986. 10.1002/cam4.155 24403272 PMC3892403

[4] Farquhar DR, Divaris K, Mazul AL, Weissler MC, Zevallos JP, Olshan AF. Poor oral health affects survival in head and neck cancer. Oral Oncol 2017; 73: 111–117. 10.1016/j.oraloncology.2017.08.009 28939062 PMC5659716

[5] Baudin F, Guillot P, Bedock B, Blanc PL. Refractory thrombotic thrombocytopenic purpura revealing an epiglotis neoplasia. Ann Fr Anesth Reanim 2012; 31: 478–480. 10.1016/j.annfar.2012.01.035 22465649

[6] Yang CW, Wang SF, Yang XL, Wang L, Niu L, Liu JX. Identification of gene expression models for laryngeal squamous cell carcinoma using co-expression network analysis. Medicine (Baltimore) 2018; 97: e9738. 29443735 10.1097/MD.0000000000009738 PMC5839854

[7] Kara M, Uysal S, Altinisik U, Cevizci S, Guclu O and Derekoy FS. The pre-treatment neutrophil-to-lymphocyte ratio, platelet-to-lymphocyte ratio, and red cell distribution width predict prognosis in patients with laryngeal carcinoma. Eur Arch Otorhinolaryngol 2017; 274: 535–542. 10.1007/s00405-016-4250-8 27520567

[8] Huang L, Wang S, Li SS, Yang XM. Prognostic significance of Beclin-1 expression in laryngeal squamous cell carcinoma. Pathol Oncol Res 2013; 19: 771–777. 10.1007/s12253-013-9642-0 23686476

[9] Zhang W, Shen Y. Platelet-to-Lymphocyte Ratio as a New Predictive Index of Neurological Outcomes in Patients with Acute Intracranial Hemorrhage: A Retrospective Study. Med Sci Monit 2018; 24: 4413–4420. 10.12659/MSM.910845 29946059 PMC6052826

[10] Vernieri C, Mennitto A, Prisciandaro M, Huber V, Milano M, Rinaldi L, et al The neutrophil-to-lymphocyte and platelet-to-lymphocyte ratios predict efficacy of platinum-based chemotherapy in patients with metastatic triple negative breast cancer. Sci Rep 2018; 8: 8703 10.1038/s41598-018-27075-z 29880896 PMC5992181

[11] Chen L, Zeng H, Yang J, Lu Y, Zhang D, Wang J, et al Survival and prognostic analysis of preoperative inflammatory markers in patients undergoing surgical resection for laryngeal squamous cell carcinoma. BMC Cancer 2018; 18: 816 10.1186/s12885-018-4730-x 30103707 PMC6090788

[12] Schariatzadeh R, Pezier TF, Studer G, Schmid S, Huber GF. Does airway intervention before primary nonsurgical therapy for T3/T4 laryngeal squamous cell carcinoma impact on oncological or functional outcomes? Swiss Med Wkly 2015; 145: w14213 10.4414/smw.2015.14213 26715377

[13] Yang L, Dong H, Li Z, Pan Y, Qu L, Tan Z. Correlation between circulating tumor cells and D-D and platelet in patients with pulmonary malignancies. Oncol Lett 2018; 15: 2169–2172. 10.3892/ol.2017.7595 29434921 PMC5776933

[14] Wei T, Zhang LN, Lv Y, Ma XY, Zhi L, Liu C, et al Overexpression of platelet-derived growth factor receptor alpha promotes tumor progression and indicates poor prognosis in hepatocellular carcinoma. Oncotarget 2014; 5: 10307–10317. 10.18632/oncotarget.2537 25333264 PMC4279374

[15] Jiang L, Luan Y, Miao X, Sun C, Li K, Huang Z, et al Platelet releasate promotes breast cancer growth and angiogenesis via VEGF-integrin cooperative signalling. Br J Cancer 2017; 117: 695–703. 10.1038/bjc.2017.214 28697175 PMC5572171

[16] Lee KI, Olmer M, Baek J, D'Lima DD, Lotz MK. Platelet-derived growth factor-coated decellularized meniscus scaffold for integrative healing of meniscus tears. Acta Biomater 2018; 76: 126–134. 10.1016/j.actbio.2018.06.021 29908335 PMC6090559

[17] Griffin RJ, Williams BW, Wild R, Cherrington JM, Park H, Song CW. Simultaneous inhibition of the receptor kinase activity of vascular endothelial, fibroblast, and platelet-derived growth factors suppresses tumor growth and enhances tumor radiation response. Cancer Res 2002; 62: 1702–1706. 11912143

[18] Zhang LX, Wei ZJ, Xu AM, Zang JH. Can the neutrophil-lymphocyte ratio and platelet-lymphocyte ratio be beneficial in predicting lymph node metastasis and promising prognostic markers of gastric cancer patients? Tumor maker retrospective study. Int J Surg 2018; 56: 320–327. 10.1016/j.ijsu.2018.06.037 29969732

[19] Viers BR, Thompson RH, Lohse CM, Cheville JC, Leibovich BC, Boorjian SA, et al Pre-treatment neutrophil-to-lymphocyte ratio predicts tumor pathology in newly diagnosed renal tumors. World J Urol 2016; 34: 1693–1699. 10.1007/s00345-016-1821-7 27052014

[20] Morizawa Y, Miyake M, Shimada K, Hori S, Tatsumi Y, Nakai Y, et al Neutrophil-to-lymphocyte ratio as a detection marker of tumor recurrence in patients with muscle-invasive bladder cancer after radical cystectomy. Urol Oncol 2016; 34: 257 e211–257. 10.1016/j.urolonc.2016.02.012 27038696

[21] Alexander ET, Minton AR, Peters MC, van Ryn J, Gilmour SK. Thrombin inhibition and cisplatin block tumor progression in ovarian cancer by alleviating the immunosuppressive microenvironment. Oncotarget 2016; 7: 85291–85305. 10.18632/oncotarget.13300 27852034 PMC5356737

[22] Sudoyo AW, Rachman A, Harimurti K. Angiogenesis, inflammation, platelets count, and metastatic status as a predictor for thrombosis risk in nasopharyngeal carcinoma patients. Acta Med Indones 2015; 47: 11–15. 25948762

[23] Zhu GS, Tian SB, Wang H, Ma MG, Liu Y, Du HS, et al Preoperative Neutrophil Lymphocyte Ratio and Platelet Lymphocyte Ratio Cannot Predict Lymph Node Metastasis and Prognosis in Patients with Early Gastric Cancer: a Single Institution Investigation in China. Curr Med Sci 2018; 38: 78–84. 10.1007/s11596-018-1849-6 30074155

[24] Xu F, Xu P, Cui W, Gong W, Wei Y, Liu B, et al Neutrophil-to-lymphocyte and platelet-to-lymphocyte ratios may aid in identifying patients with non-small cell lung cancer and predicting Tumor-Node-Metastasis stages. Oncol Lett 2018; 16: 483–490. 10.3892/ol.2018.8644 29928436 PMC6006354

[25] Caine GJ, Ryan P, Lip GY, Blann AD. Significant decrease in angiopoietin-1 and angiopoietin-2 after radical prostatectomy in prostate cancer patients. Cancer Lett 2007; 251: 296–301. 10.1016/j.canlet.2006.11.026 17240049

[26] Dong L, Bai K, Cao Y, Huang Q, Lv L, Jiang Y. Prognostic Value of Pre-Operative Platelet to Lymphocyte Ratio in Patients with Resected Primary Hepatocellular Carcinoma. Clin Lab 2016; 62: 2191–2196. 10.7754/Clin.Lab.2016.160414 28164677

[27] Temur I, Kucukgoz Gulec U, Paydas S, Guzel AB, Sucu M, Vardar MA. Prognostic value of pre-operative neutrophil/lymphocyte ratio, monocyte count, mean platelet volume, and platelet/lymphocyte ratio in endometrial cancer. Eur J Obstet Gynecol Reprod Biol 2018; 226: 25–29. 10.1016/j.ejogrb.2018.05.028 29804024

[28] Seropian IM, Romeo FJ, Pizarro R, Vulcano NO, Posatini RA, Marenchino RG, et al Neutrophil-to-lymphocyte ratio and platelet-to-lymphocyte ratio as predictors of survival after heart transplantation. ESC Heart Fail 2018; 5: 149–156. 10.1002/ehf2.12199 28758719 PMC5793982

[29] Dong H, Ren JX, Wang JJ, Ding LS, Zhao JJ, Liu SY, et al Chinese Medicinal Leech: Ethnopharmacology, Phytochemistry, and Pharmacological Activities. Evid Based Complement Alternat Med 2016; 2016: 7895935 10.1155/2016/7895935 27274755 PMC4870366

