# Correction: May the change of platelet to lymphocyte ratio be a prognostic factor for T3-T4 laryngeal squamous cell carcinoma: A retrospective study

**DOI:** 10.1371/journal.pone.0226383

**Published:** 2019-12-05

**Authors:** Bing Zhong, De-Ying Gu, Jin-Tao Du, Fei Chen, Ya-Feng Liu, Shi-Xi Liu

There are multiple errors in the “Results” subsection of the Abstract. The correct paragraph is: “The 413 consecutive patients with LSCC were reviewed. Of these, 362 patients who met the criteria were selected, multi-factor analysis found that pathological T classification (hazard ratio [HR] = 1.878; 95% confidence interval [CI] = 1.342–3.023; P<0.001), pathological N classification (HR = 1.212; 95% CI = 1.167–2.125; P< 0.001) and change of PLR (HR = 2.158; 95% CI = 1.332–2.889; P = 0.003) associated with postoperative recurrence of T3-T4 LSCC. In addition, the pathological T classification (HR = 1.901; 95% CI = 1.255–2.999; P<0.001), pathological N classification (HR = 1.244; 95% CI = 1.110–2.212; P<0.001) and change of PLR (HR = 2.011; 95% CI = 1.354–2.753; P = 0.001) associated with postoperative survival in patients with T3-T4 LSCC.”

There is an error in the second sentence of the second paragraph of the Results. The correct sentence is: “Multivariate analyses showed that pathological T classification (HR = 1.878; 95% CI = 1.342–3.023; P<0.001), pathological N classification (HR = 1.212; 95% CI = 1.167–2.125; P<0.001) and change of PLR (HR = 2.158; 95% CI = 1.332–2.889; P = 0.003) were independent risk factors for a worsened RFS (Table 3).”

There is an error in the second sentence of the third paragraph of the Results. The correct sentence is: “Pathological T classification (HR = 1.901; 95% CI = 1.255–2.999; P<0.001), pathological N classification (HR = 1.244; 95% CI = 1.110–2.212; P<0.001) and change of PLR (HR = 2.011; 95% CI = 1.354–2.753; P = 0.001) were demonstrated as independent prognostic factors for poor OS rate in the multivariate analysis (Table 5).”

In Table 3, the Pathological T stage, Pathological N stage, and Change of PLR variables list incorrect HR, 95% CI, and P values. Please see the correct Table 3 here.

**Table 3:** Factors associated with postoperative recurrence in the multivariate analyses.

Variables	HR	95% CI	P
Pathological T stage	1.878	1.342–3.023	<0.001
Pathological N stage	1.212	1.167–2.125	<0.001
Change of PLR	2.158	1.332–2.889	0.003

In Table 5, the Pathological T stage, Pathological N stage, and Change of PLR variables list incorrect HR, 95% CI, and P values. Please see the correct Table 5 here.

**Table 5:** Factors associated with postoperative survival in the multivariate analyses.

Variables	HR	95% CI	P
Pathological T stage	1.901	1.255–2.999	<0.001
Pathological N stage	1.244	1.110–2.212	<0.001
Change of PLR	2.011	1.354–2.753	0.001
